# When COVID‐19 delays the management of an urgent heart condition: A rare case of a spontaneous dissection of two coronary arteries

**DOI:** 10.1002/ccr3.4708

**Published:** 2021-08-30

**Authors:** Gislain Beyina Endamena, Mazou Ngou Temgoua, Sylvain Chanseaume, Enver Hilic, Lise Camus, Alexandra Chanseaume, Alexandru Mischie, Karamoko Kane, Nouhoun Diallo, Sami Assi, Romain Eschalier

**Affiliations:** ^1^ Cardiology Unit Center Hospital of Monluçon Monluçon France; ^2^ Department of Internal Medicine and Specialities Faculty of Medicine and Biomedical Sciences Yaoundé Cameroon; ^3^ Department of Cardiology Clermont‐Ferrand University Hospital Clermont‐Ferrand France

**Keywords:** COVID‐19, delay management, spontaneous coronary arteries dissection

## Abstract

Some severe life‐threatening conditions could be misdiagnosed during the current COVID‐19 pandemic.

## Graphical abstract

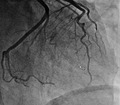

## 1 INTRODUCTION

COVID‐19 is a worldwide crisis with a great impact in health structures. Delay in the management of routine medical conditions has been reported during this pandemic. We describe the case of a spontaneous dissection of two coronary arteries which has been initially misclassified as a case of COVID‐19 infection and managed lately.

Spontaneous coronary artery dissection (SCAD) is a rare cause of acute coronary syndrome (ACS) and sudden death. It is defined as a separation within the coronary artery wall secondary to intramural hemorrhage, with or without tearing of the intima, thus modifying arterial architecture with the creation of a true and false channels. The starting point is often an intimal breach or even a hemorrhage of the vasa vasorum.^1^ SCAD preferentially affects the young population with almost no traditional cardiovascular risk factors and especially female with a sex ratio M/F of 1/5.^2^ It is responsible for 0.1%–0.4% of all acute coronary syndromes.^3^ It is an important cause of ACS in women and represents approximately 25% of acute coronary syndromes in women under 50 years old.^4^ It has the same clinical presentation as acute coronary syndromes secondary to plaque ruptures. Many triggering factors are mentioned in the occurrence of a SCAD including physical or emotional stress.^5^

Currently, the whole world experiences a very stressful condition generated by COVID‐19 pandemic. Since the beginning of this pandemic, healthcare facilities precisely emergency room are overcrowded. So, with that massive influx of patients, many other diseases are misdiagnosed.^6^ Xiang et al. in their study analyzing the management and outcomes of patients with ST segment elevation myocardial infarction during the COVID‐19 pandemic in China found an important decrease of the hospitalized patient with ST segment elevation myocardial infarction still the outbreak of COVID‐19 pandemic. Also, they found a delay in management that patients according to the reperfusion strategy.^6^ Similarly, Rashid et al. found a decline of acute myocardial infarction‐related hospitalization associated with a raise in the number of cases of out‐of‐hospital cardiac arrest since the beginning of this pandemic, particularly after the implementation of social confinement measures during the COVID‐19 outbreak in England. Consequently, the reorganization of hospital services and medical staff in response to COVID‐19 outbreak affected routine management of other diseases.^7^ Through this case, we wanted to show the harmful influence of the current pandemic in the management of potential life‐threatening issue as SCAD.

## 2 CASE PRESENTATION

**FIGURE 1:**
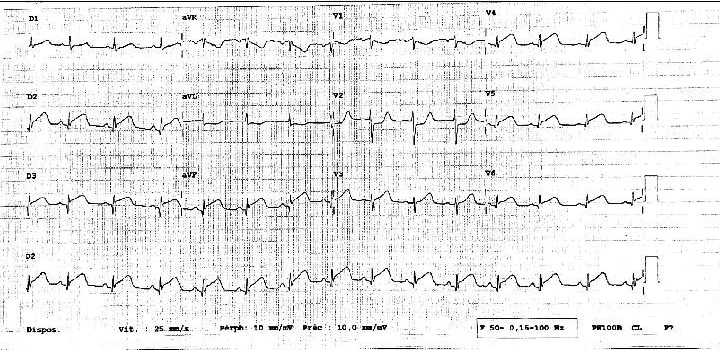
12‐Limbs electrocardiogram showing an infero‐lateral ST segment elevation

**FIGURE 2:**
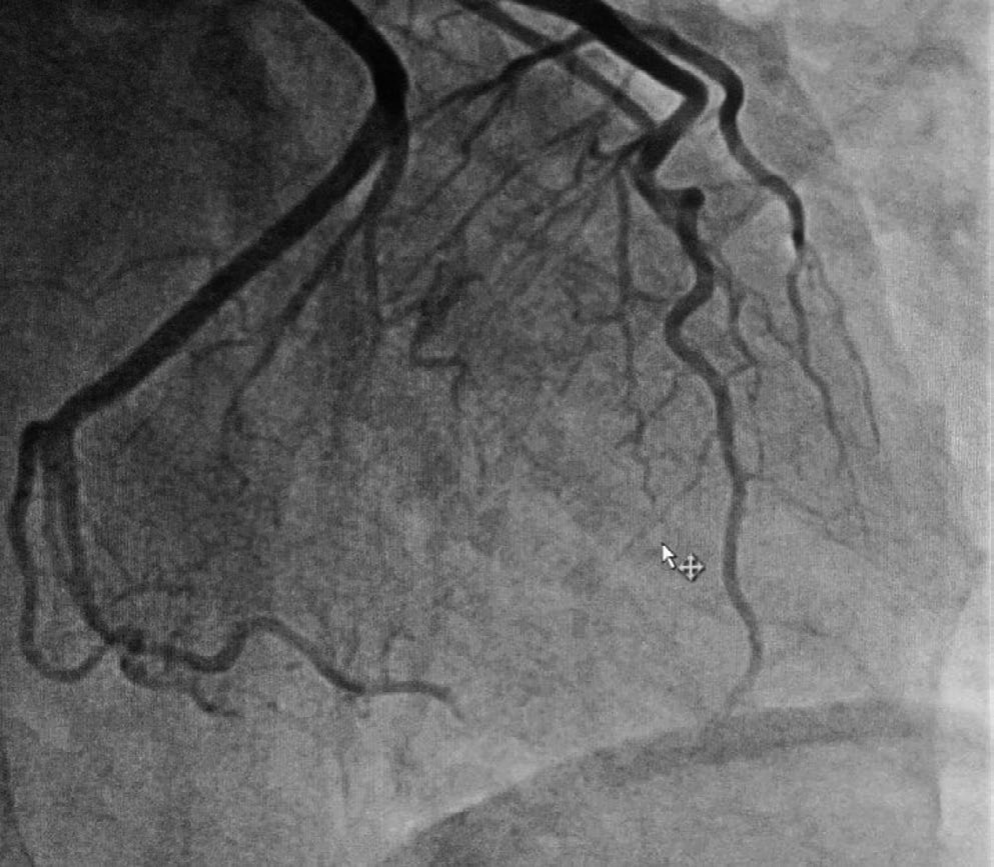
Coronary artery image showing a type 1 spontaneous dissection of the distal interventricular artery and a type 2 dissection of the second marginal

This is a 61‐year‐old patient, an active smoker at 20 pack‐years, in full professional activity during the period of total confinement, has presented a presyncope in the morning around 09.50 am without chest pain, or loss of consciousness or fall with notion of chills a few hours earlier. She was transported by the firefighters at 10:30 am for the Montluçon Hospital Center. The patient was received around 11:40 am and referred to its sector hospital (Gueret Hospital Center) in accordance with the COVID‐19 Plan from the Regional Health Agency for screening as suspected of coronavirus infection 19. On arrival at the emergency room of this health facility, an electrocardiogram carried out revealed an apicolateral and inferior persistent ST segment elevation with mirror image on anterior area (Figure 1). Then, the patient has been transported to the initial center for emergency coronary angiography where she arrived at 3:30 pm. Coronary angiography was performed and revealed the presence of a thrombus in the distal anterior interventricular artery with the appearance of "stick insect," a thrombus on the dominant circumflex artery obstructing the second marginal with a "radish tail" aspect and a right network free from any atheromatous lesion (Figure 2). Transthoracic cardiac ultrasound found good biventricular systolo‐diastolic function with a left ventricular ejection fraction of 69%, no abnormalities in segmental and global kinetics of the left ventricle. Troponin I was 2465 pg/ml, Creatine phosphokinase at 1874 pg/ml, C‐reactive protein 1.1 mg/L, leukocytes at 13,81.10^9^/L. The medical treatment included glycoprotein IIb / IIIa inhibitor, double anti‐aggregation platelet and anticoagulant (unfractionated heparin), beta‐blocker (bisoprolol) on Day 1 hospitalization. Bisoprolol has been changed to ivabradine because the patient remained tachycardic with low blood pressure. We noted on the Day 3 electrocardiogram, presence of a Q wave of apicolateral and inferior necrosis with persistence of the elevation from the same territory. Treatment with acetylsalicylic acid, clopidogrel, ivabradine, and inhibitor of the proton pump was given as an exit treatment. Magnetic resonance imaging (MRI) was performed on an outpatient basis at one month and found: a sequelae of infero‐latero‐moderate, infero‐septo‐average, latero‐apical, infero‐apical, and septo‐apical with thinning of the middle and apical walls measuring, respectively, 3mm and 4mm, a left ventricular ejection fraction of 47%.

**FIGURE 3:**
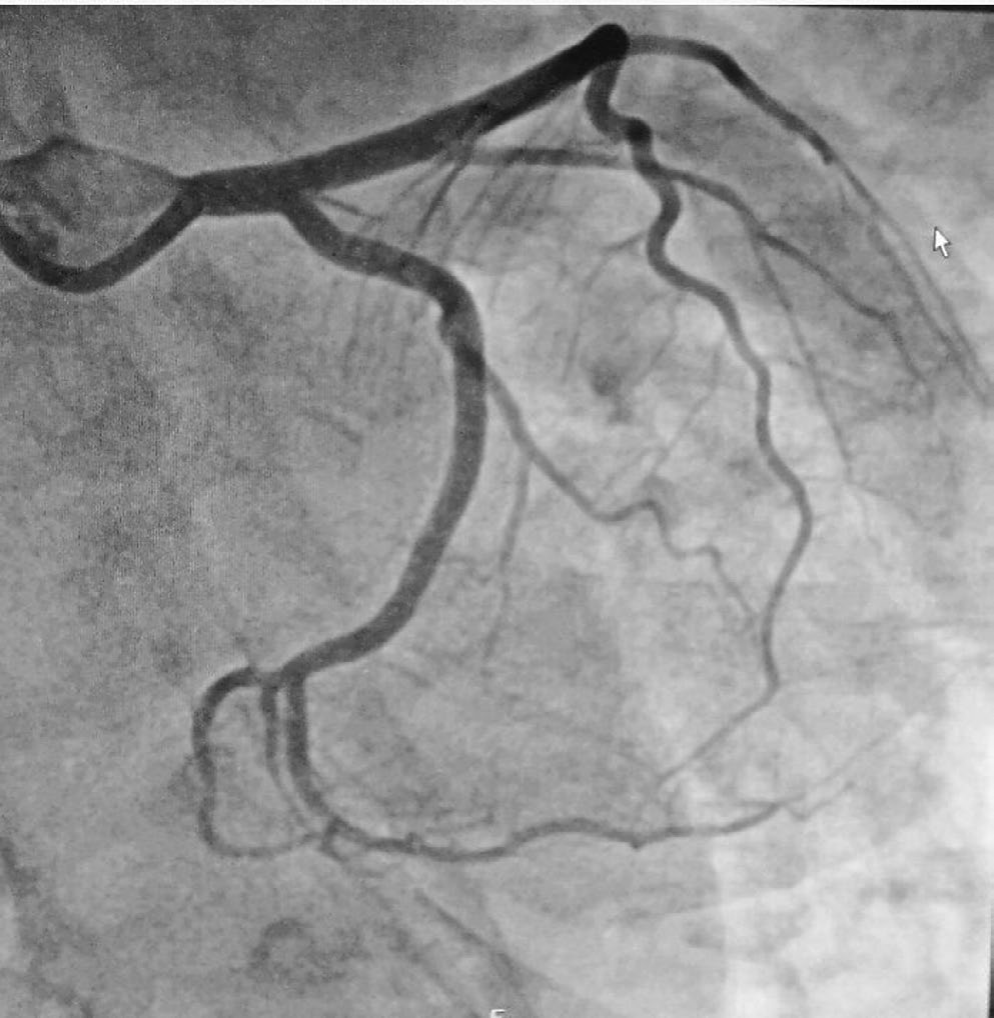
Coronarographic control after 2 months

**FIGURE 4:**
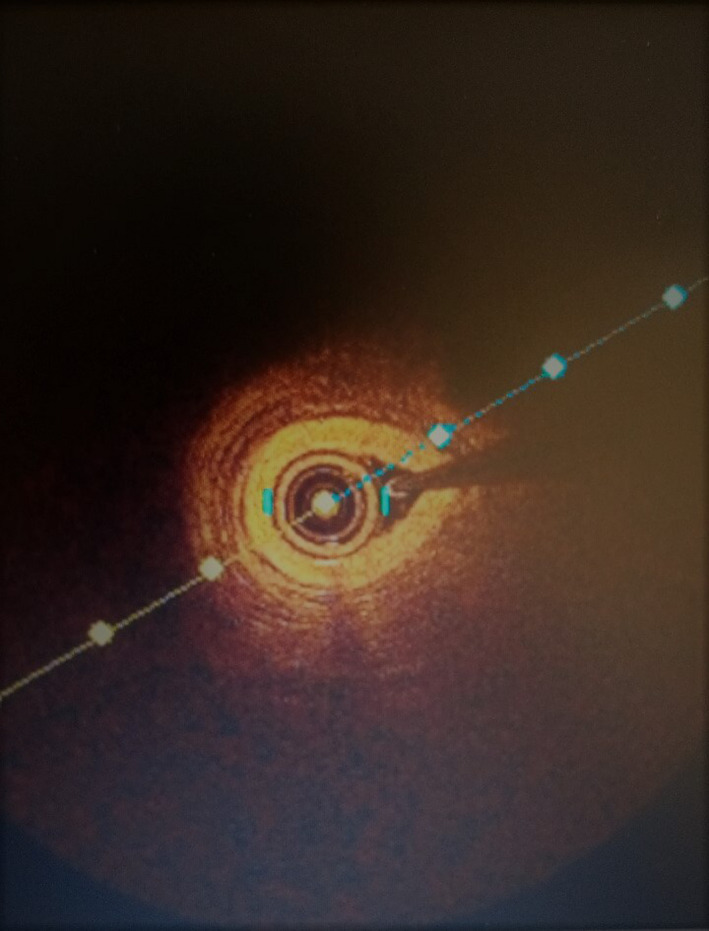
Optical coherence tomography image

Coronarography control with optical coherence tomography (OCT) performed two months later allowed to highlight a complete healing of the lesions (Figure 3). OCT does not highlighted the intimal breach (Figure 4).

## 3 DISCUSSION

Formerly assimilated to a classic acute coronary syndrome, the diagnosis of SCAD was based mainly on the presence of an intimal radiolucent flap associated with a contrast in the arterial wall on coronary angiography. Currently, studies on SCAD permit to establish a classification into 3 angiographic types^8^: Type 1 (representing the classic description with intimal flap), type 2 (extensive and diffuse tubular lesions with a plane of dissection not visible which may result in complete coronary occlusion), and type 3 (multiple focal tubular lesions due to intramural hematoma mimicking atherosclerosis).

Our patient presented a feature of a double dissection with a type 1 lesion on the distal anterior interventricular artery and a type 2 lesion on the second left marginal. Type 2, sometimes resulting in compression of the vascular lumen due to intramural hematoma without intimal flap, it is the most frequently encountered in the different series representing 67% of cases. Type 1 follow‐up with an intimal flap which represents 29% of cases. Type 3 mimicking atherosclerotic lesions is only found in 4% of cases.^8^ The descending anterior interventricular artery is usually the most affected during SCAD. However, polyarterial lesions could be found in a range of 20%−25% of cases.^9^

Patients with SCAD generally have arterial fragility without atheroma or calcifications which may limit the progression of coronary dissection. This fragility can be acquired during pregnancy, oral contraception, hormone therapy, systemic inflammatory diseases, congenital condition as in fibromuscular dysplasia, Marfan disease, syndrome Ehlers‐Danlos, connectivitis, or even idiopathic.^10^, ^11^ In the series by Saw et al assessing the baseline characteristics, predisposing factors, and clinical outcomes of 168 patients with a SCAD, the association with a detailed screening of other non‐coronary arterial disease had revealed that 72% of these patients also presented with fibromuscular dysplasia.^5^ However, our patient did not present clinical elements that could suggest any of the above conditions cited. These changes in the arterial wall are generally not sufficient to explain SCAD. This pathology appears to be multifactorial. An extrinsic trigger appears essential, such as emotional stress, intense physical exercise, or maneuvers such as Valsalva.^10^, ^11^ This is also mentioned in the series by Saw et al. In this study, physical or emotional stress was implicated as triggering factors in 56% of cases.^5^ Likewise, in our patient, we could clearly evoke an emotional and important professional stress. In fact, concomitantly with the occurrence of this case of spontaneous dissection of two coronary arteries, we were living a period of general confinement in France due to the COVID‐19 pandemic. Very anxiety‐provoking situation both professional and personal for our patient who continue to carry out her professional activities despite the high risk of contracting a particularly fatal viral disease. We therefore think that the strong stress at the same time emotional, physical, and professional would be the triggering factor. Direct link between SARS‐COV2 and SCAD is not clear but as there is a strong relationship between cardiovascular disease and COVID‐19, future studies are needed in this field.^12^ The clinical presentation of a SCAD is similar to that of an acute coronary syndrome by rupture atherosclerotic plaque. In more than half of the cases, we note electrocardiographic changes as ST segment elevation associated with elevation of enzymes myocardial (troponin). The rest of the cases will present as acute non‐ST elevation coronary syndromes.^13^

Some may also present with short‐term life‐threatening ventricular arrhythmias and others still in cardiac arrest. In our case, we had a typical electrocardiographic presentation of a myocardial infarction with infero‐lateral ST segment elevation and an anterior mirror image. Of course, we had an enzymatic movement with elevation of troponins I. In the study done by the Mayo Clinic in the United States of America with the highest number of patients with spontaneous coronary arteries dissection, aimed at determining the prevalence of coronary tortuosity in these patients, it appears that tortuosity of coronary arteries is a risk factor for SCAD and even recurrence.^13^

As part of the SCAD, coronary angiography remains the first‐line examination and sometimes allows to visualize thrombi in the real channel, but it makes it difficult to visualize hematoma of the artery wall. It also helps to determine the type of lesion. It is therefore systematically recommended an association with intravascular imaging as optical coherence tomography (OCT) or intravascular ultrasonography (IVUS). The optical coherence tomography has better resolution than intravacular ultrasonography. It allows to confirm the guide's position in the real channel, detail the site of the wall hematoma and intimal breach, to facilitate an accurate assessment of the size of the artery and optimize stent expansion in the event of underlying angioplasty.^14^ However, OCT therefore allows a detailed overview of the arterial anatomy of patients.^15^ This technique is limited by its lack of visualizing deep layers of the arterial wall.^15^ Compared to intravascular ultrasound, OCT is clearly superior in terms of the location of the initial intimal lesion and in the exploration of the intimo‐medial membrane (intimal flap).^15^, ^16^

The treatment of SCAD could be medical generally without stenting and the prognostic is generally good.^14^ Our treatment consists of an antiplatelet drug, a beta‐blocker in order to reduce the pressure shearing action on the arterial wall and thus stop intramural bleeding favorizing the repair of intimal breach. The myocardial sequelae were found after one month in our patient probably because of the association of two coronary arteries involvement and the delay of management. Although these sequelae have disappeared after two months with good medical therapy.

## 4 CONCLUSION

Spontaneous dissection of coronary arteries is a rare cause of acute coronary syndrome. As for takotsubo cardiomyopathy, it should always be evoked in young female with thoracic pain in the context of severe stress. The health practitioners should be more alerted especially during this current pandemic.

## CONFLICT OF INTEREST

None.

## AUTHOR CONTRIBUTIONS

GBE: Manuscript writing. MNT: Critical revision. RE: Supervision. All the authors: Management of the case.

## ETHICAL APPROVAL

Formal ethical approval from the University Research Ethics Board was not required for the completion of this study.

## INFORMED CONSENT

Written informed consent for publication of this case report was obtained from the patient.

## Data Availability

The data that support the findings of this study are available from the corresponding author upon reasonable request.
